# Distinct BAL cytokine signatures define immunopathological endotypes in interstitial lung disease

**DOI:** 10.3389/fphys.2026.1951891

**Published:** 2026-10-13

**Authors:** Rubén Osuna-Gómez, Ana Pardessus, Maria Mulet, Marta Hernández Marcos, Mᵅ Àngels Ortiz, Virginia Pajares, Elisabet Cantó, Joel Francesqui, José Manuel Brenes, Albert Guinart-Cuadra, Silvia Vidal, Diego Castillo

**Affiliations:** 1 Group of Inflammatory Diseases, Institut de Recerca Sant Pau (IR Sant Pau), Barcelona, Spain; 2 Respiratory Department, Hospital de la Santa Creu i Sant Pau, Barcelona, Spain; 3 Radiology Department, Hospital Santa Creu i Sant Pau, Universitat Autónoma Barcelona (UAB), Barcelona, Spain

**Keywords:** bronchoalveolar lavage, cluster analysis, cytokines, fibrosis, inflammatory profile, interstitial lung disease

## Abstract

**Background:**

Interstitial lung disease (ILD) comprises a heterogeneous group of disorders characterized by variable clinical phenotypes and dysregulated immune responses. The marked diversity in disease behavior and therapeutic response suggests the existence of distinct molecular endotypes. In this study, we applied an unsupervised clustering analysis to bronchoalveolar lavage (BAL) cytokines to identify clinically meaningful ILD subpopulations.

**Methods:**

BAL cytokine data from ILD patients were analyzed using K-means clustering, identifying four primary clusters: (C1) TGF-β/IL-17A dominant, (C2) IL-6 dominant, (C3) IL-35/IL-10 dominant, and (C4) lacking a dominant cytokine signature. Immune cell composition, clinical characteristics, and disease progression were compared across clusters.

**Results:**

Clusters C1 and C2 were associated with greater fibrotic burden. C1, characterized by elevated TGF-β and IL-17A levels, showed a higher prevalence of idiopathic pulmonary fibrosis (IPF) and increased BAL macrophages. C2, with high IL-6, included a higher proportion of smoke-related ILD (SR-ILD) and was associated with increased cardiovascular comorbidities, hospitalizations, and BAL neutrophils. In contrast, C3 and C4, with higher IL-35 and IL-10, were associated with lower fibrosis severity and a greater proportion of connective tissue disease-associated ILD (CTD-ILD). C3 and C4 showed more favorable clinical outcomes and disease stability over one year; notably, only C3 maintained stability over two years and exhibited increased BAL CD4+ T cells.

**Conclusion:**

Clustering of BAL cytokine profiles revealed distinct immunological endotypes in ILD that correlated with fibrosis severity, clinical phenotype, and disease trajectory. This approach holds promise for improving patient stratification and prediction of disease progression in ILD.

## 1 Introduction

Interstitial lung disease (ILD) comprises a variety of diffuse parenchymal lung disorders characterized by different patterns of the inflammation and fibrosis of the lung (Sellarés and Molina-Molina, 2020; Wijsenbeek and Cottin, 2020). In a significant proportion of cases, ILD follows a chronic course marked by progressive scarring of lung tissue (Althobiani et al., 2024). Idiopathic Pulmonary Fibrosis (IPF) is the paradigm of chronic fibrotic ILD, with a median survival of 3–5 years after diagnosis (Chen et al., 2021). Currently, no curative therapy exists for pulmonary fibrosis. Antifibrotic agents, such as pirfenidone and nintedanib, slow disease progression in some patients but show variable efficacy and tolerability (Khan et al., 2023). Identifying patients who will benefit most from these treatments in routine clinical practice remains challenging.

Accumulating evidence suggests that an altered immune response is involved in ILD pathogenesis, with various immune subsets potentially contributing to the development of this disease, particularly macrophages and lymphoid cells (Osuna-Gómez et al., 2023; Tseng et al., 2023; Karaselek et al., 2024). During fibrogenesis, epithelial cells undergoing apoptosis and activated immune cells, such as macrophages, promote aberrant tissue repair through the release of proinflammatory and profibrotic mediators, including transforming growth factor beta (TGF-β) and IL-6 (Confalonieri et al., 2022; Ge et al., 2024). These cytokines have been demonstrated to have a central profibrotic remodeling in the lung and also promote T helper-17 (Th17) cell differentiation (Simonian et al., 2009; Wang et al., 2024). Th17 lymphocytes secrete IL-17, a proinflammatory cytokine that has been shown to exacerbate pulmonary fibrosis in a bleomycin-induced murine model (Lei et al., 2016).

Recent reports have suggested the context dependent role of regulatory T cells (Tregs) in pulmonary fibrosis (Seyran et al., 2023). While Tregs may promote fibrosis through TGF-β secretion (Chen et al., 2024), they can also exert anti-fibrotic effects through a variety of mechanisms, such as the secretion of IL-10 or IL-35 (Nakagome et al., 2006; Collison et al., 2010; Osuna-Gómez et al., 2023). IL-35 promotes the proliferation and differentiation of IL-35-producing Tregs, which inhibits effector T cell activation and Th17-mediated inflammation (Wang and Lei, 2021). On the other hand, IL-10 and IL-35 have been reported to attenuate pulmonary fibrosis in a TGF-β1-dependent manner (Nakagome et al., 2006; Hu et al., 2020). These findings underscore the dual and tightly regulated roles of immune pathways in fibrotic lung disease.

Despite growing evidence of immune dysregulation in ILD, most studies have relied on single-marker approaches, limiting our understanding of immune interactions underlying disease complexity of ILD (Wang et al., 2023). Clustering analysis offer a powerful data-driven framework to integrate multiple biomarkers simultaneously, thereby enabling the identification of immunologically distinct endotypes within heterogeneous datasets (McDermott et al., 2013; Dalmaijer et al., 2022).

The diversity of clinical presentation, disease progression, and treatment response in ILD suggests the presence of distinct molecular and immunological subpopulations within ILD (Kraven et al., 2023). Prior studies have indicated the potential existence of ILD endotypes based on clinical and biomarker profiles (Jenkins et al., 2015; Maher et al., 2017; Fainberg et al., 2022). Previous work has found that cluster analysis can effectively identify endotypes in ILD, integrating various data sources including clinical and blood biomarker data (Fainberg et al., 2024). Additionally, epithelial dysfunction profiles have been described at varying stages of IPF progression (Jenkins et al., 2015; Maher et al., 2017) further supporting the concept of disease endotyping However, immunological endotypes derived directly from the pulmonary compartment remain insufficiently characterized.

Given the profound heterogeneity of ILD and the central role of immune-mediated mechanisms in fibrosis, we hypothesized that distinct bronchoalveolar lavage (BAL) cytokine profiles reflect specific immune pathways that define clinically relevant ILD endotypes. To test this hypothesis, we (i) quantified key profibrotic and immunoregulatory cytokine (TGF-β, IL-35, IL-10, IL-17A, and IL-6) in BAL samples from ILD patients, (ii) applied *K*-means clustering to identify cytokine-based ILD endotypes, and (iii) examined associations between these clusters, immune cell populations, clinical characteristics, and disease progression. Collectively, this approach aims to immunological distinct ILD endotypes that may improve patient stratification and guide future therapeutic strategies.

## 2 Materials and methods

### 2.1 Design

This cluster analysis was performed using bronchoalveolar lavage (BAL) samples obtained from the Sant Pau Pulmonary Fibrosis Biobank. This collection gathers clinical data, BAL and biological samples from consecutively new diagnosed ILD patients. The Sant Pau Pulmonary Fibrosis collection is registered and approved by the Spanish National Research Institute (registration number C.0005695). All patients provided written consent for inclusion in the biobank. Ethical approval for this study was granted by the Hospital de la Santa Creu i Sant Pau Institutional Ethics Committee.

### 2.2 Population

The study population consisted of newly diagnosed patients referred to the ILD clinic between 2014 to 2019, in whom BAL was performed as part of their diagnostic assessment. Patients were managed according to the current recommendations by their physicians, independently of their participation in this study. Diagnoses were confirmed by a multidisciplinary ILD team in accordance with current guidelines (Travis et al., 2013; Raghu et al., 2018). Patients were excluded if they had received antifibrotic or immunomodulatory therapies prior to BAL collection.

Cases were classified as fibrotic ILD (F-ILD) or non-fibrotic ILD (NF-ILD) (Osuna-Gómez et al., 2023; Osuna-Gómez et al., 2024) based on visual analysis of chest high-resolution computed tomography (HRCT). Those cases with radiological evidence of fibrosis (defined as reticulation, traction bronchiectasis or honeycombing) affecting more than 5% of lung parenchyma were classified as F-ILD.

### 2.3 Data collection

Follow-up clinical data at 12 and 24 months and survival were available for all patients. For each patient, demographic and clinical variables were collected at baseline, including age, sex, smoking status, comorbidities, symptoms (dyspnea and cough), radiological pattern on HRCT scan, BAL immune cell subpopulations determined by flow cytometry, lung function test (LFT)- including diffusing capacity for carbon monoxide (DLCO) and forced vital capacity (FVC)- and diagnosis. In addition, treatment recommended at the multidisciplinary ILD meeting was also recorded.

To monitor progress, the following variables were gathered at 12 and 24 months after diagnosis: symptoms (dyspnea and cough), LFT, chest HRCT, hospitalization and death. An absolute decline of ≥5% in FVC and/or ≥10% in DLCO was established as a significant change in LFT (Macaluso et al., 2022). Dyspnea severity was assessed in all patients using the Medical Research Council (MRC) dyspnea scale (Bestall et al., 1999). This scale grades breathlessness from 0 to 4 based on the level of physical activity that provokes symptoms, with grade 4 indicating dyspnea during minimal exertion. To assess the long-term prognosis, vital status was recorded from the last appointment available.

### 2.4 Processing of BAL

Bronchoscopy with BAL was performed according to the European Respiratory Society BAL Task Force Group guidelines (Clinical guidelines and indications for bronchoalveolar lavage (BAL): Report of the European Society of Pneumology Task Group on BAL, 1990). BAL fluid was filtered through sterile gauze and centrifuged at 600 × g for 10 min at room temperature. The supernatant was aliquoted and stored at −80 °C until analysis.

For immunophenotyping, BAL cells were stained with the following monoclonal antibodies: anti-CD3-FITC (Immunotools Friesoythe, Germany), anti-CD4-Viogreen (Miltenyi Biotec, Bergisch Gladbach, Germany), anti-CD56-PE (Immunotools), anti-CD14-PE-Vio615 (Miltenyi Biotec), anti-CD45-PE-Cy7 (BD Biosciences, San Jose, USA), anti-CD8-APC (Immunotools), and anti-CD20-APC-Cy7 (Miltenyi Biotec). CD45+ cells were gated to identify leukocytes. Within the lymphocyte gate, CD4+ T cells were defined as CD3+CD4+CD8−, CD8+ T cells as CD3+CD4−CD8+, and B cells as CD3−CD20+. Macrophages were identified based on forward scatter (FSC), side scatter (SSC), and CD14 expression. Neutrophils were identified by FSC/SSC characteristics and CD16 positivity, while eosinophils were defined as CD16−, as previously described by our group (Osuna-Gómez et al., 2024). Data acquisition was performed on a MACSQuant Analyzer 10 flow cytometer (Miltenyi Biotec) and analysis was conducted with FlowJo version 10 (FlowJo LLC, Ashland, OR, USA).

### 2.5 Determination of BAL cytokines

BAL concentrations of IL-35 (Cloud-Clone Corp, Wuhan, China), TGF-β (Mabtech, Nacka Strand, Sweden), IL-10 (Immunotools), IL-17A (PeproTech EC, London, UK), and IL-6 (Immunotools) from ILD patients were determined using commercially available ELISA kits according to the manufacturers’ instructions. Cytokine concentrations were calculated using standard curves generated with recombinant molecules. The limits of detection were as follows: 1.56–100 pg/ml for IL-35, 62.5–4000 pg/mL for TGF-β, 15.62–1000 pg/mL for IL-10, 15.62–1000 pg/mL for IL-17A, and 8–500 pg/ml for IL-6. Cytokine concentrations were expressed as raw (uncorrected) values per milliliter of BAL fluid (pg/mL).

### 2.6 Clustering analysis

Prior to clustering, the distribution of variables was assessed using the Kolmogorov-Smirnov test. Variable independence was evaluated through bivariate correlation analyses and visual inspection of scatter plots. To reduce redundancy and improve model performance, variables with strong correlations (r > 0.6 and p < 0.05) were excluded.

Exploratory heatmaps based on BAL cytokine concentrations (TGF-β, IL-35, IL-10, IL-17A, and IL-6) were generated using R software (v 4.3.1) to visualize potential patient groupings. Subsequently, *K*-means clustering was performed using JASP version 0.17.2 software (Amsterdam, The Netherlands). The Hartigan–Wong algorithm was applied to minimize within-cluster variance and maximize between-cluster separation (Kamali and Stashuk, 2020; Wang et al., 2021).

The optimal number of clusters was determined using the elbow method, which evaluates the proportion of variance explained across different cluster solutions (Payton et al., 2022). Cluster quality was further assessed using the Silhouette coefficient, which quantifies the degree of separation between clusters (Dinh et al., 2019). Cluster stability was assessed using R software (v 4.3.1) with the clusterboot function, based on 1,000 bootstrap resampling iterations. Stability was quantified using the Jaccard similarity coefficient, with higher values indicating more stable clusters (Hennig, 2007).

### 2.7 Statistics

Statistical analyses were performed using GraphPad Prism version 10 (GraphPad Software, La Jolla, CA, USA). Normal data distribution was assessed using the Kolmogorov–Smirnov test. Continuous variables were presented as mean ± SEM for normally distributed data or median (interquartile range, IQR) for non-normally distributed data. Comparisons among more than two groups were performed using one-way analysis of variance (ANOVA) with Bonferroni correction (parametric data) or the Kruskal–Wallis test with Dunn’s *post hoc* correction (non-parametric data). Chi-square test were used for the comparison of frequencies. Correlation analyses were determined with Pearson’s or Spearman’s correlation according to normal distribution. Overall survival was estimated using the Kaplan–Meier method and analyzed using the log-rank test. Overall survival was defined as the interval from diagnosis to death or last follow-up. Multivariate survival analysis was performed using Firth’s penalized likelihood Cox proportional hazards regression (coxphf package) in R software (v4.3.1) to evaluate the prognostic independence of the cytokine clusters while adjusting for age, sex, baseline FVC (%), and underlying disease etiology (IPF, CTD-ILD, and others). A p-value < 0.05 was considered statistically significant.

## 3 Results

### 3.1 Demographics

We included 63 cases. Demographic and clinical characteristics are presented in **Table 1**. The mean age was 64 years and there was a predominance of male patients (35 cases). A total of 36 cases (57.1%) were classified as F-ILD. The most frequent diagnoses were connective tissue disease-associated ILD (CTD-ILD) (23.8%), followed by IPF (17.5%). Oxygen therapy was prescribed in nearly 19% of the patients. A third of the patients (31.7%) were initiated on treatment after the multidisciplinary ILD meeting.

**Table 1:** Patient characteristics.

Characteristics	Category	Value
Median age (range), years		64 (26-86)
Male/Female		35/28
Fibrotic status, n (%)	Fibrotic	36 (57.1%)
	Non-fibrotic	27 (42.9%)
Smoke status, n (%)	Current	22 (34.9%)
	Never	16 (25.4%)
	Ex-smoker	25 (39.7%)
Comorbidities, n (%)	Cancer	4 (6.3%)
	Cardiovascular disease	17 (27%)
Exacerbations, (%)		15.9%
Oxygen therapy, (%)		19%
Initial DLCO, (%)		59.9%
Initial FVC, (%)		82.9%
Etiology, (%)	IPF	17.5%
	CTD-ILD	23.8%
	U-ILD	11.1%
	SR-ILD	15.9%
	HP	7.9%
	Sarcoidosis	12.7%
	PLCH	11.1%
Treatment, (%)	NT	68.3%
	AF	6.3%
	IS	25.4%

DLCO, diffusing capacity for carbon monoxide; FVC, forced vital capacity; IPF, idiopathic pulmonary fibrosis; CTD, connective tissue disease-associated; ILD, U, unclassifiable ILD; HP, hypersensitivity pneumonitis; SC, sarcoidosis; PLCH, pulmonary langerhans cell histiocytosis; SR, smoking-related ILD; NT, non-treated; AF, anti-fibrotic therapy; IS, immunosuppressors.

### 3.2 Cluster classification

To explore overall cytokine expression profiles across ILD cases, a heatmap of normalized BAL cytokine concentrations (TGF-β, IL-35, IL-10, IL-17A, and IL-6) was generated. This visualization suggested the presence of heterogeneous cytokine profiles among patients (**Figure 1A**).

**Figure 1:**
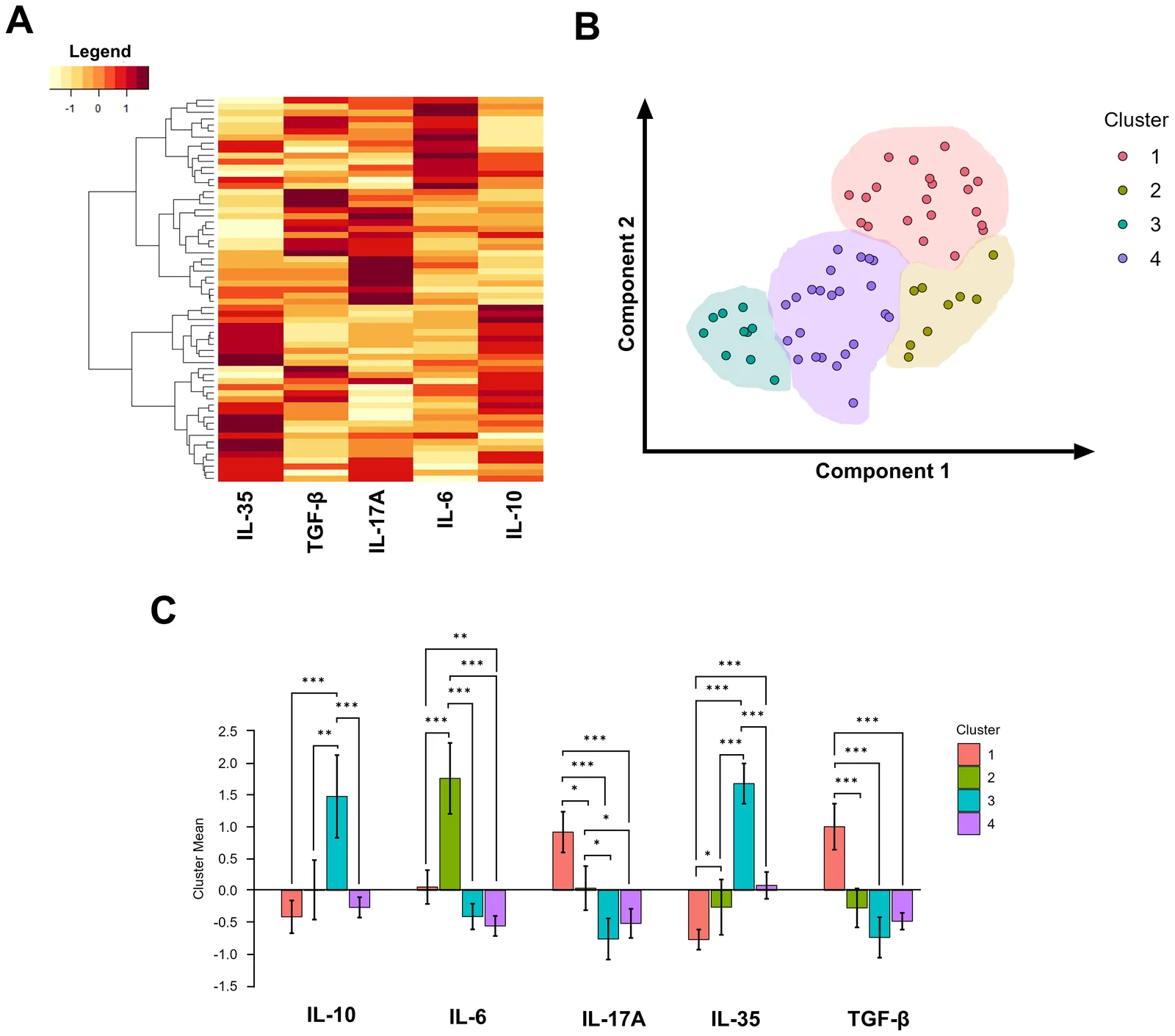
Cytokine-based clustering analysis of ILD patients. **(A)** Heatmap showing the distribution of ILD patients based on BAL cytokine concentrations (IL-35, IL-10, IL-6, TGF-β, IL-17A). Each column represents a cytokine, and each row corresponds to an individual patient. Colors represent the scaled expression values, with red indicating higher expression and yellow indicating lower expression relative to the overall cohort average. **(B)** *K*-means clustering of ILD patients using cytokine concentration data. The plot shows the distribution of patients between four distinct clusters (C1–C4), represented by different colors (red, yellow, blue, and purple), along principal component axes. **(C)** Comparative profile of BAL cytokine levels within patient clusters. The y-axis represents the cluster mean, which indicates whether the cytokine levels in each cluster are above (positive values) or below (negative values) the overall cohort mean value. Adjusted p-values were obtained using Bonferroni or Dunn’s test corrections, according to their normal distribution. *p < 0.05; **p < 0.01; ***p < 0.001.

After confirming that the BAL cytokines did not exhibit significant multicollinearity, these variables were included in a *K*-means clustering analysis to identify distinct endotypes based on cytokine concentrations. The optimal number of clusters (*k* = 4) was determined using the elbow method, and cluster quality (0.52) was determined by the Silhouette coefficient. Bootstrap resampling and Jaccard similarity coefficient (0.85 for C1, 0.77 for C2, 0.84 for C3, and 0.78 for C4), indicated high cluster stability (> 0.75). The resulting clusters were as follows: Cluster 1 (C1) n = 21, Cluster 2 (C2) n = 10, Cluster 3 (C3) n = 23, and Cluster 4 (C4) (n = 9). As shown, the resulting four clusters defined distinct cytokine-based ILD endotypes (**Figure 1B**). C1 was characterized by high TGF-β and IL-17A together with low IL-35 concentrations. C2 showed high IL-6 with intermediate concentrations of the other cytokines. C3 displayed high IL-10 and IL-35 but low TGF-β concentrations, while C4 exhibited relatively high IL-35 but not IL-10 concentrations (**Figure 1C**; **Supplementary Table S1**).

### 3.3 Clinical characteristics associated with clusters

We first analyzed the clinical characteristics across clusters. Patient age was comparable among clusters (**Figure 2A**). C1 and C2 had a higher percentage of patients with cardiovascular comorbidities (**Figure 2B**). C2 also had a higher percentage of patients requiring hospitalization during follow-up (**Figure 2C**).

**Figure 2:**
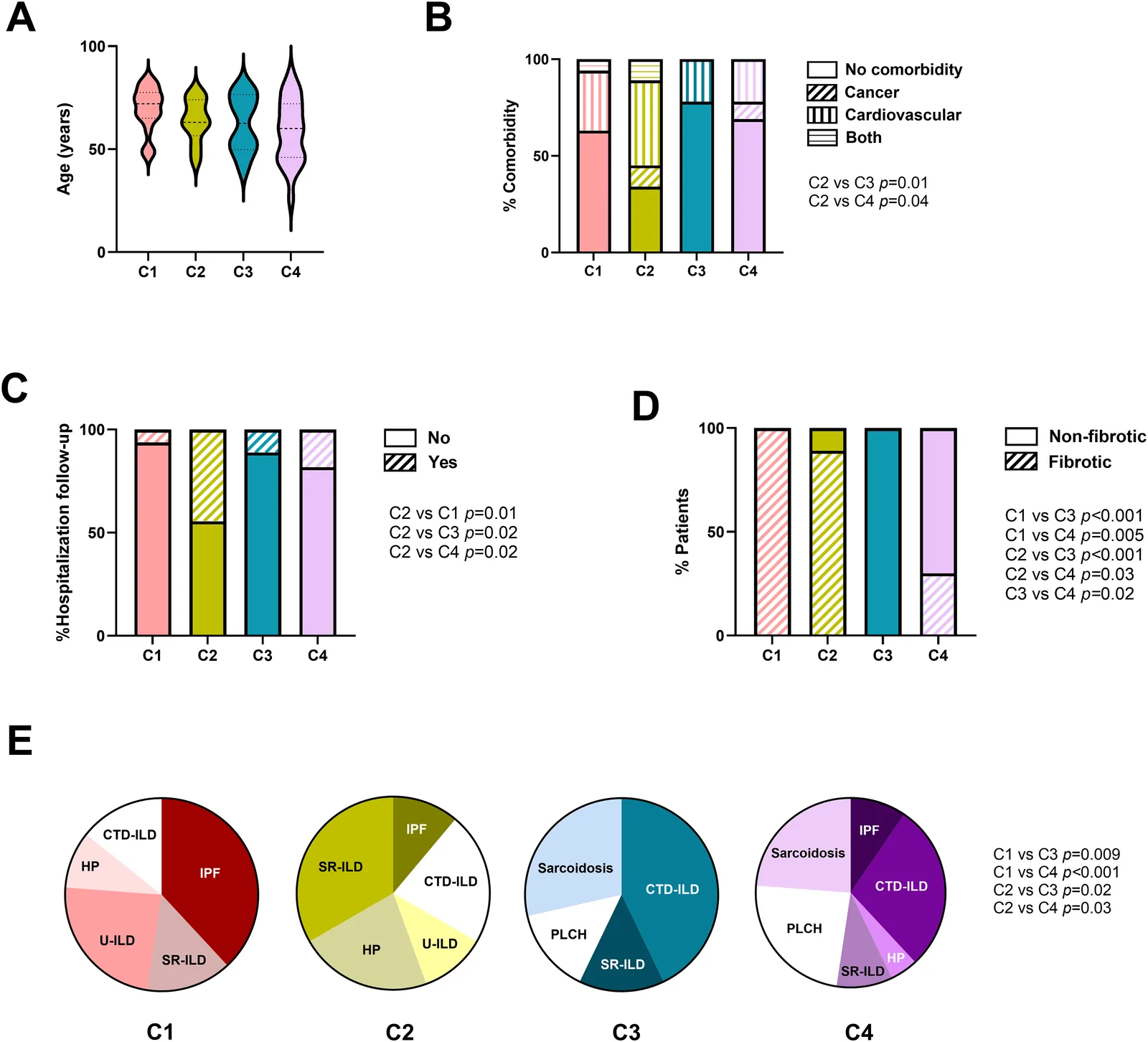
Comparison of baseline features across ILD patient clusters. **(A)** Comparison of mean age between clusters. Adjusted p-values were obtained using Bonferroni test corrections, according to their normal distribution. Comparative analysis of the percentage of patients **(B)** with various comorbidities, **(C)** severe exacerbations that required hospitalizations, and **(D)** the presence of fibrosis between clusters. **(E)** Comparative analysis of etiology between clusters (IPF, idiopathic pulmonary fibrosis; CTD, connective tissue disease-associated ILD; U, unclassifiable ILD; HP, hypersensitivity pneumonitis; PLCH, pulmonary langerhans cell histiocytosis; and SR, smoking-related ILD). p-value were obtained using the Chi-square test. Clusters were represented by different colors (C1: red; C2: yellow; C3: blue; and C4: purple).

C1 and C2 had a higher percentage of patients with fibrotic ILD compared with C3 and C4, with C3 showing the lowest percentage of fibrotic cases (**Figure 2D**). Regarding ILD classification, C1 had the highest percentage of patients with IPF and unclassifiable ILD (U-ILD), whereas C2 had the highest percentage of patients with SR-ILD and hypersensitivity pneumonitis (HP). C3 had the highest percentage of sarcoidosis and CTD-ILD, whereas C4 had the highest percentage of patients with pulmonary Langerhans cell histiocytosis (PLCH) (**Figure 2E**). The distribution of CTD-ILD subtypes across clusters is shown in **Supplementary Table S2**. All four CTD-ILD subtypes were represented across the four clusters. C3 had the highest proportion of patients with CTD-ILD (9/23), comprising 3 patients with Sjögren syndrome, 4 with systemic sclerosis, 1 with myositis, and 1 with rheumatoid arthritis.

C3 had better preserved lung function compared to the other clusters (**Figures 3A, B**). Analysis of BAL myeloid cells revealed that C1 had more percentage of macrophages than C2 or C3, whereas C2 had the highest percentage of neutrophils (**Figures 3C, D**). Percentages of eosinophils were comparable across clusters (**Figure 3E**). Within the lymphoid compartment, C3 had a higher percentage of CD4+ T cells, with the consequent lower percentages of CD8+ T cells compared with the other clusters (**Figures 3F, G**). B-cell percentages were comparable among clusters (**Figure 3H**).

**Figure 3:**
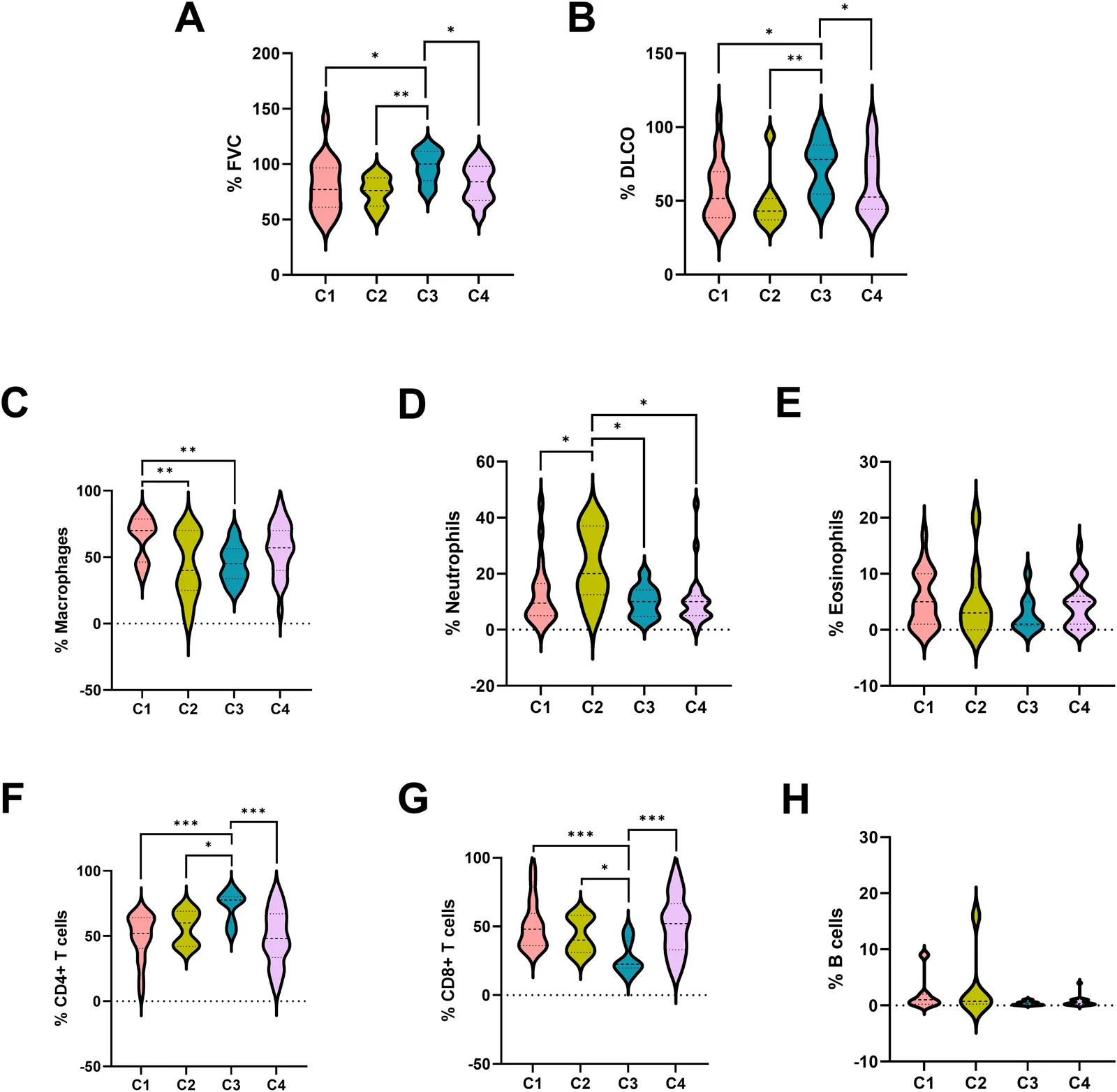
Comparison of lung function and immune cell profiles in BAL across ILD patient clusters. Comparison of the percentage of patients with different levels of **(A)** diffusing capacity for carbon monoxide (DLCO), and **(B)** forced vital capacity (FVC) between clusters. **(C–H**) Comparative analysis of immune cell percentages in BAL: **(C)** macrophages, **(D)** neutrophils, **(E)** eosinophils, **(F)** CD4+ T cells, **(G)** CD8+ T cells, and **(H)** B cells between clusters. Adjusted p-values were obtained using Bonferroni or Dunn’s test corrections, according to their normal distribution. Clusters were represented by different colors (C1: red; C2: yellow; C3: blue; and C4: purple). *p < 0.05; **p < 0.01; ***p < 0.001.

To evaluate how these cytokine-defined endotypes align with conventional clinical stratifications, we performed a complementary analysis evaluating the relative distribution of C1–C4 clusters by binary clinical categories: fibrotic versus non-fibrotic ILD, and IPF versus non-IPF ILD (**Supplementary Table S3**). Cluster distribution differed significantly between fibrotic and non-fibrotic ILD, with C1 and C2 predominantly comprising fibrotic patients (58.3% and 22.2%, respectively) and C3 predominantly representing non-fibrotic ILD (85.1%). Similarly, cluster distribution differed significantly between IPF and non-IPF status, with C1 being markedly enriched in IPF cases (72.7%) and C3 in non-IPF cases (44.2%).

### 3.4 Clinical features of disease progression

Disease progression was analyzed across clusters. After one year of follow-up, C3 had a higher percentage of patients with stable FVC whereas C1 and C2 had a higher percentage of patients with FVC decline. After two years of follow-up, this pattern persisted, with C3 maintaining the highest percentage of patients with stable FVC (**Figure 4A**). After one year all clusters had comparable proportions of patients with DLCO decline. At two years of follow-up, C4 had a lower percentage of patients with stable DLCO compared with C2 and C3 (**Figure 4B**).

**Figure 4:**
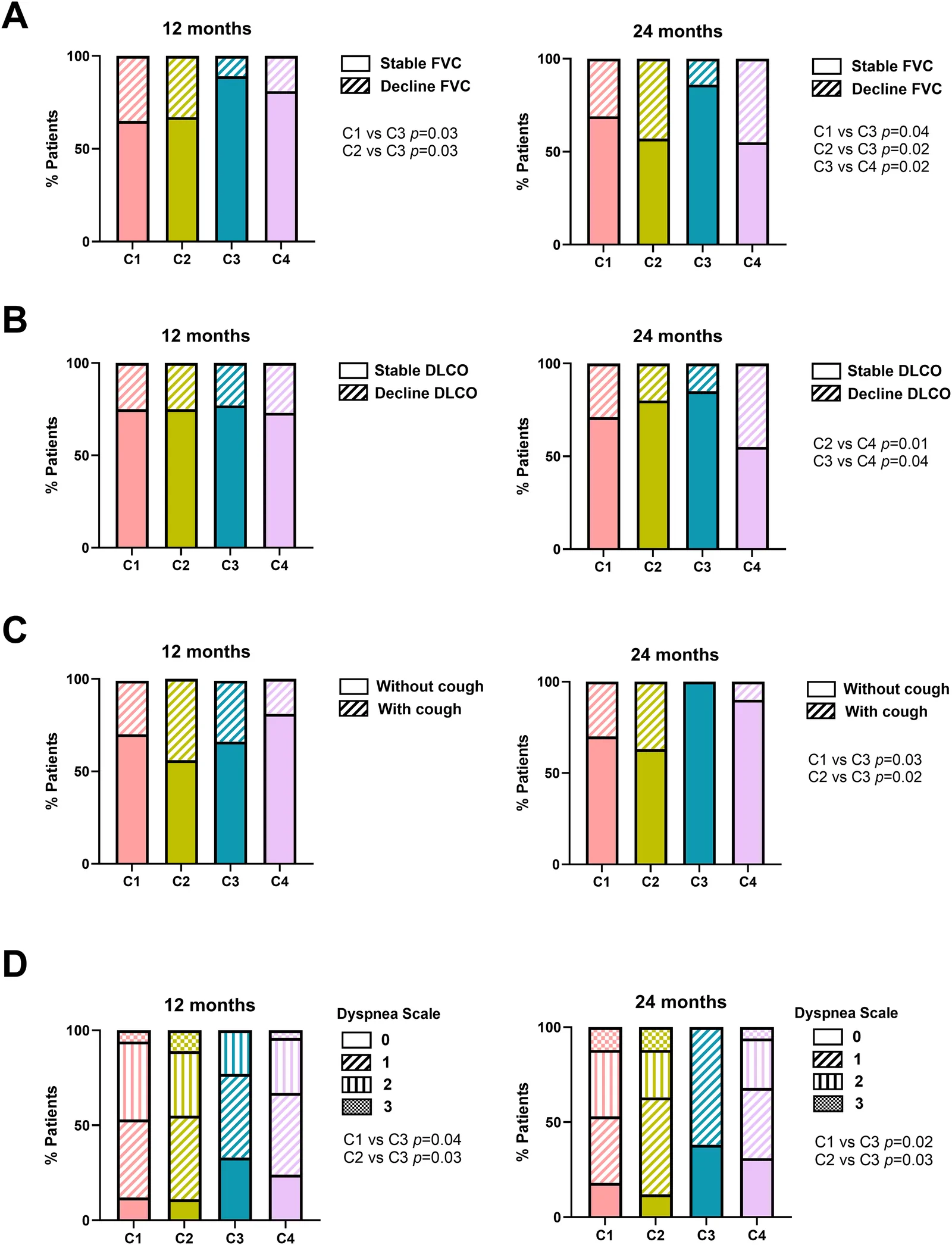
Comparison of progression after one- and two-years follow-up among ILD patient clusters. Comparative analysis of the percentage of patients with stable or worsened **(A)** forced vital capacity (FVC) or **(B)** diffusing capacity for carbon monoxide (DLCO) progression between clusters. Comparison of the percentage of patients according to **(C)** the presence of cough or **(D)** dyspnea scale grade between clusters. Clusters were represented by different colors (C1: red; C2: yellow; C3: blue; and C4: purple). p-value were obtained by Chi-square test.

After one year of follow-up, the percentage of patients with cough was similar across clusters. However, after two years, the percentage of patients with cough was higher in C1 and C2 than in C3 (**Figure 4C**). C1 and C2 also had a higher percentage of patients with severe dyspnea compared with C3 at both one- and two-year follow-up (**Figure 4D**).

We also evaluated these functional and symptomatic progression endpoints using binary ILD stratifications: fibrotic versus non-fibrotic ILD, and IPF versus non-IPF ILD (**Supplementary Table S4**). No significant differences were observed in FVC or DLCO decline at 12 or 24 months according to either fibrotic status or IPF diagnosis. In contrast, the proportion of patients with cough at 24 months was significantly higher in fibrotic ILD compared with non-fibrotic cases, as well as in IPF compared with non-IPF patients. Additionally, severe dyspnea at 24 months was significantly more frequent in fibrotic ILD than in non-fibrotic ILD, whereas no differences were observed based on IPF status.

### 3.5 Clinical outcomes

To analyze a possible effect of treatment for clinical outcomes, we evaluated different treatments between clusters. No significant differences were observed between clusters in the percentages of patients receiving immunosuppressants, antifibrotic drugs, or no treatment (**Figure 5A**). When evaluating radiologic progression at one-year follow-up, we observed a higher percentage of worsening disease in C2 patients compared with the other clusters. C1 and C4 patients also showed higher rates of radiological progression than C3, in which all patients exhibited stable disease (**Figure 5B**).

**Figure 5:**
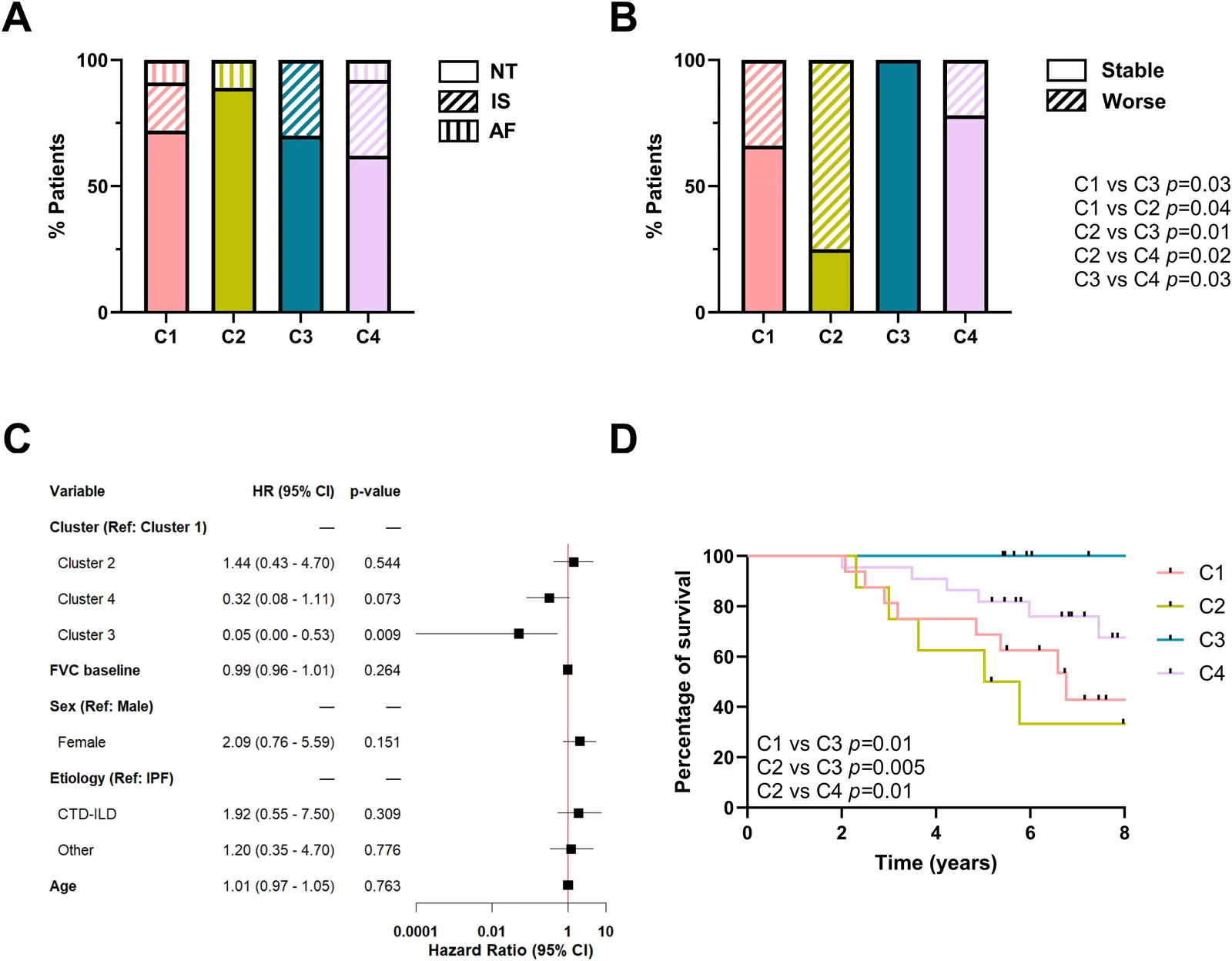
Comparison of survival across ILD patient clusters. **(A)** Comparative analysis of the percentage of patients receiving different treatments between clusters (NT: non-treated; IS: immunosuppressors; AF: antifibrotics). p-value were obtained by Chi-square test. **(B)** Comparative analysis of the percentage of patients with stable or worsened radiologic progression at one-year follow-up. p-value were obtained by Chi-square test. **(C)** Forest plot of the multivariate Firth’s penalized Cox regression model. Hazard ratios (HR) and 95% profile likelihood confidence intervals for cytokine clusters and clinical covariates (age, sex, baseline FVC, and underlying etiology) were represented. **(D)** Kaplan–Meier survival curves were used to compare survival between clusters, and differences in survival were assessed using the log-rank test. Clusters were represented by different colors (C1: red; C2: yellow; C3: blue; and C4: purple).

Then, using Kaplan–Meier survival analysis, we found better overall survival in C3 and C4 compared with C1 or C2, with the most favorable prognosis in C3 (**Figure 5D**). Median overall survival was 6.6 years for C1, 5.0 years for C2, 8.0 years for C3, and 7.2 years for C4.

To determine whether the prognostic value of the cytokine clustering was independent of clinical diagnoses, a multivariate Firth’s penalized Cox model was performed (**Figure 5C**). After adjusting for age, sex, baseline FVC, and etiology, C3 remained a statistically significant independent protective factor against mortality. Cluster 4 showed a similar protective trend, though it did not reach statistical significance. The clinical covariates were not statistically significant.

Finally, we assessed radiological progression, treatment distribution, and overall survival based on the same binary ILD classifications (**Supplementary Table S4**). Radiological worsening at 12 months was significantly more frequent in patients with fibrotic ILD than in those with non-fibrotic ILD, whereas no significant differences were observed according to IPF status. Treatment distribution was comparable across groups under both binary stratifications. Regarding overall survival, Kaplan–Meier analysis revealed a significantly lower survival rate for patients with fibrotic compared to those with non-fibrotic disease (**Supplementary Figure S1A**), whereas IPF status did not show a statistically significant impact on long-term outcomes (**Supplementary Figure S1B**).

## 4 Discussion

This study identifies distinct immunological endotypes of ILD based on cytokine concentrations (TGF-β, IL-17A, IL-6, IL-10, and IL-35) in BAL. By applying *K*-means clustering, we defined four cytokine-based groups (C1–C4) with distinct immune profiles and clinical characteristics. C1 was characterized by elevated TGF-β and IL-17A concentrations, and was associated with fibrotic ILD forms and poorer clinical outcomes. In contrast, C2 showed predominant IL-6 upregulation with neutrophilic inflammation, consistent with an inflammatory endotype linked to disease exacerbations and adverse outcomes. C3 exhibited high IL-10 and IL-35 concentrations, reflecting an immunoregulatory profile associated with preserved lung function and improved survival. C4 displayed increased IL-35 without concomitant IL-10 elevation, corresponding to an intermediate clinical phenotype. Collectively, these findings demonstrate that BAL cytokine profiling identifies immunological distinct ILD endotypes with potential clinical relevance.

The clustering revealed a close association between IL-17A and TGF-β, both key mediators of fibrogenesis, suggesting a pivotal role in fibrosis progression, particularly in C1. Similarly, the IL-6-dominant profile found in C2 aligns with its established role in inflammatory amplification and neutrophil recruitment, supporting the presence of an aggressive inflammatory endotype (Dufour et al., 2018). In contrast, the concurrent elevation of IL-35 and IL-10 suggests a strongly immunoregulatory environment (Pan et al., 2019; Steen et al., 2020), whereas C4, distinguished by elevated IL-35 alone may reflect partial immune regulation through distinct mechanisms.

Baseline clinical features differed among clusters, corroborating the association between cytokine profiles and disease phenotype. C1 showed greater fibrosis, aligning with the elevated levels of TGF-β and IL-17A (Sun et al., 2018; Wakhlu et al., 2018), and a higher prevalence of IPF and U-ILD, and increased macrophage representation in BAL, consistent with a profibrotic immune milieu (Ying et al., 2021). C2 was enriched in SR-ILD and HP and exhibited higher rates of cardiovascular complications and hospitalizations, reflecting a more unstable and inflammatory disease course supported by neutrophil predominance in BAL (Tezcan et al., 2021; Lee et al., 2021). C3 included more cases of sarcoidosis and CTD-ILD, showed better preserved lung function, and had higher proportions of CD4+ T cells, suggesting a balanced immune response protective against disease progression (Nakagome et al., 2006; Osuna-Gómez et al., 2023). C4 exhibited intermediate outcomes and was enriched in PLCH, supporting its classification as a non-fibrotic but incompletely regulated endotype. Together, these observations underscore that BAL cytokine profiles mirror the underlying immune–pathological mechanisms in ILD and provide a framework for linking immune pathways with clinical features.

Longitudinal follow-up reinforced these associations C1 showed the most pronounced functional decline, with a greater reduction in FVC and DLCO values and poorer overall survival. These patients also had a higher frequency of severe dyspnea and cough, reflecting a progressive fibrotic phenotype associated with unfavorable clinical outcomes. C2, with an inflammatory profile and higher burden of fibrotic ILDs, displayed the most aggressive disease course, with rapid deterioration of lung function and poor overall survival, and with higher hospitalization rates. Previous studies have linked elevated plasmatic IL-6 levels to poor prognosis in ILD (De Lauretis et al., 2013; Lee et al., 2021). Interestingly, tocilizumab (IgG1 humanized anti-IL-6 receptor monoclonal antibody) has been shown to stabilize FVC independent of fibrosis severity in systemic sclerosis patients with ILD (Roofeh et al., 2021). C3 maintained stable lung function over time and exhibited the best survival outcomes, paralleling observations in other chronic lung diseases where decreased levels of IL-10 and IL-35 are associated with disease progression (Jiang et al., 2018). C4 exhibited an intermediate clinical trajectory, characterized by structural rather than fibrotic damage. Taken together, these findings indicate that BAL cytokine profiles could reflect clinically meaningful ILD endotypes with differential clinical outcomes.

This cluster-based approach may contribute to precision medicine in ILD by stratifying patients according to their underlying biological profiles in addition to established clinical features. In the future, such molecular stratification could help identify patient subgroups more likely to benefit from specific therapeutic strategies, potentially enabling a more individualized approach to treatment selection.

Several limitations should be acknowledged. The relatively small sample size, particularly within C2 and C4, may also limit the robustness and generalizability of the identified endotypes. Cytokine levels were not assessed in plasma, precluding direct comparison between systemic and pulmonary compartments. Additionally, the current approach does not allow assessment of cytokine modulation in response to therapy. Larger cohorts and longitudinal analyses during treatment will be required to validate these cytokine-based endotypes and explore their utility as biomarkers of disease progression or treatment response.

In conclusion, this study demonstrates that BAL cytokine clustering identifies biologically and clinically meaningful ILD endotypes. These findings emphasize the potential of immune-based stratification to improve prognostic assessment and support the development of more personalized therapeutic strategies in ILD.

## Data Availability

The raw data supporting the conclusions of this article will be made available by the authors, without undue reservation.
