# Supplementary material for: Distinct BAL cytokine signatures define immunopathological endotypes in interstitial lung disease
Source: Front Physiol. 2026 Oct 13;17:1951891. doi: 10.3389/fphys.2026.1951891 (PMC13710069; doi:10.3389/fphys.2026.1951891)
Supplement: Supplementary file 1 [file Table1.docx]

**Supplementary material**

**Table S1.** BAL cytokine concentrations in the different clusters

| **Cytokine (Mean±SEM)** | **C1** | **C2** | **C3** | **C4** | **­*p*-value** |
| --- | --- | --- | --- | --- | --- |
| TGF-β (pg/ml) | 468±40 | 234±33 | 149±35 | 196±14 | C1 vs C2 p=0.001 |
|  |  |  |  |  | C1 vs C3 p<0.001 |
|  |  |  |  |  | C1 vs C4 p<0.001 |
| IL-35 (pg/ml) | 17.5±1.4 | 25.1±3.9 | 53.8±2.8 | 30.1±1.8 | C1 vs C2 p=0.03 |
|  |  |  |  |  | C1 vs C3 p<0.001 |
|  |  |  |  |  | C1 vs C4 p<0.001 |
|  |  |  |  |  | C2 vs C3 p<0.001 |
|  |  |  |  |  | C3 vs C4 p<0.001 |
| IL-6 (pg/ml) | 17.6±1.8 | 36.8±3.8 | 12.3±1.3 | 10.7±1.0 | C1 vs C2 p<0.001 |
|  |  |  |  |  | C1 vs C4 p=0.002 |
|  |  |  |  |  | C2 vs C3 p<0.001 |
|  |  |  |  |  | C2 vs C4 p<0.001 |
| IL-17 (pg/ml) | 10.5±0.5 | 8.14±0.5 | 5.98±0.5 | 6.64±0.3 | C1 vs C2 p=0.01 |
|  |  |  |  |  | C1 vs C3 p<0.001 |
|  |  |  |  |  | C1 vs C4 p<0.001 |
|  |  |  |  |  | C2 vs C3 p=0.01 |
|  |  |  |  |  | C2 vs C4 p=0.04 |
| IL-10 (pg/ml) | 9.49±0.7 | 11.4±1.2 | 18.0±1.7 | 10.1±0.4 | C1 vs C3 p<0.001 |
|  |  |  |  |  | C1 vs C3 p=0.008 |
|  |  |  |  |  | C3 vs C4 p<0.001 |

Data are expressed as mean ± SEM. Adjusted p-values were obtained using Bonferroni or Dunn’s test corrections, according to their normal distribution.

**Table S2.** Distribution of CTD-ILD subtypes across the identified clusters

**Table S2.** Distribution of CTD-ILD subtypes across the identified clusters

| CTD-ILD subtype | **C1**  **(n=2)** | **C2**  **(n=2)** | **C3**  **(n=9)** | **C4**  **(n=2)** | **Total**  **(n=15)** |
| --- | --- | --- | --- | --- | --- |
| Sjögren syndrome | 50.0% | 0 % | 33.3 % | 50.0% | 33.3 % |
| Systemic sclerosis | 0 % | 50.0% | 44.5 % | 0 % | 33.3 % |
| Myositis | 50.0% | 0 % | 11.1 % | 50.0% | 20.0 % |
| Rheumatoid arthritis | 0 % | 50.0% | 11.1 % | 0 % | 13.4 % |

Percentages represent the proportion of patients within each cluster (C1-C4). CTD-ILD: connective tissue disease-associated interstitial lung disease. The total column represents the distribution of each CTD-ILD subtype in the overall study population**.**

**Table S3.** Distribution of identified clusters stratified by ILD fibrotic status and IPF diagnosis

|  | Fibrotic ILD  (n=36) | Non-fibrotic ILD  (n=27) | P value | IPF  (n=11) | Non-IPF  (n=52) | P value |
| --- | --- | --- | --- | --- | --- | --- |
| Cluster distribution, n (%) |  |  |  |  |  |  |
| C1 | 58.3 % | 0 % |  | 72.7 % | 25.0 % |  |
| C2 | 22.2 % | 3.7 % | <0.001 | 18.2 % | 15.3 % | 0.005 |
| C3 | 0 % | 85.1 % |  | 0 % | 44.2 % |  |
| C4 | 19.4 % | 11.1 % |  | 9.1 % | 15.4 % |  |

Percentages represent the distribution of patients across the four identified clusters (C1–C4) within each clinical subgroup P values were calculated using the chi-square test or Fisher's exact test for comparisons between fibrotic status (fibrotic vs non-fibrotic ILD) and IPF status (IPF vs non-IPF ILD). ILD: interstitial lung disease; IPF: idiopathic pulmonary fibrosis.

| Variable | Fibrotic ILD (n=36) | Non-fibrotic ILD  (n=27) | P value | IPF ILD  (n=11) | Non-IPF ILD (n=52) | P value |
| --- | --- | --- | --- | --- | --- | --- |
| FVC at 12 months, n (%) |  |  |  |  |  |  |
| Stable | 63.8 % | 66.7 % | n.s. | 81.8 % | 73.1 % | n.s. |
| Decline | 36.1 % | 33.3 % |  | 18.2 % | 26.9 % |  |
| FVC at 24 months, n (%) |  |  |  |  |  |  |
| Stable | 66.7 % | 66.7 % | n.s. | 72.7 % | 63.5 % | n.s. |
| Decline | 33.3 % | 33.3 % |  | 27.3 % | 36.5 % |  |
| DLCO at 12 months, n (%) |  |  |  |  |  |  |
| Stable | 77.8 % | 74.0 % | n.s. | 81.8 % | 73.0 % | n.s. |
| Decline | 22.2 % | 26.0 % |  | 18.2 % | 27.0 % |  |
| DLCO at 24 months, n (%) |  |  |  |  |  |  |
| Stable | 69.4 % | 66.7 % | n.s. | 54.5 % | 71.1 % | n.s. |
| Decline | 30.6 % | 33.3 % |  | 45.5 % | 28.9 % |  |
| Cough at 12 months, n (%) |  |  |  |  |  |  |
| No | 72.2 % | 70.4 % | n.s. | 72.7 % | 71.1 % | n.s. |
| Yes | 27.7 % | 29.6 % |  | 27.3 % | 28.9 % |  |
| Cough at 24 months, n (%) |  |  |  |  |  |  |
| No | 69.4 % | 96.3 % | 0.008 | 54.5 % | 84.6 % | 0.039 |
| Yes | 30.6 % | 3.7 % |  | 45.5 % | 15.4 % |  |
| Dyspnea score at 12 months, n (%) |  |  |  |  |  |  |
| 0 | 13.9 % | 29.6 % |  | 18.2 % | 21.1 % |  |
| 1 | 38.9 % | 48.2 % | n.s. | 63.6 % | 40.4 % | n.s. |
| 2 | 38.9 % | 22.2 % |  | 9.1 % | 34.6 % |  |
| 3 | 8.3 % | 0 % |  | 9.1 % | 3.9 % |  |
| Dyspnea score at 24 months, n (%) |  |  |  |  |  |  |
| 0 | 19.5 % | 33.3 % |  | 18.2 % | 26.9 % |  |
| 1 | 33.3 % | 51.9 % | 0.040 | 54.5 % | 38.5 % | n.s. |
| 2 | 33.3 % | 14.8 % |  | 27.3 % | 25.0 % |  |
| 3 | 13.9 % | 0 % |  | 0 % | 9.6 % |  |
| Radiological progression at 12 months, n (%) |  |  |  |  |  |  |
| Stable | 58.3 % | 85.2 % | 0.020 | 63.6 % | 71.2 % | n.s. |
| Worse | 41.6 % | 14.8 % |  | 36.4 % | 28.8 % |  |
| Treatment, n (%) |  |  |  |  |  |  |
| Antifibrotic therapy | 11.1 % | 0 % |  | 18.2 % | 3.8 % |  |
| Immunosuppressive therapy | 19.4 % | 33.3 % | n.s. | 9.1 % | 28.8 % | n.s. |
| No treatment | 69.4 % | 67.7 % |  | 72.7 % | 67.3 % |  |

**Table S4.** Clinical endpoints and treatment distribution stratified by ILD fibrotic status and IPF diagnosis

Percentages represent the proportion of patients within each clinical category (fibrotic vs non-fibrotic ILD and IPF vs non-IPF ILD). P values were calculated using the chi-square test or Fisher's exact test for comparisons between fibrotic status (fibrotic vs non-fibrotic ILD) and IPF status (IPF vs non-IPF ILD). DLCO: diffusing capacity of the lung for carbon monoxide; FVC: forced vital capacity; ILD: interstitial lung disease; IPF: idiopathic pulmonary fibrosis; n.s.: non-significant.

**Figure S1**. Kaplan-Meier survival analysis according to clinical subgroups. (**A**) Survival curves stratified by fibrotic status (fibrotic vs. non-fibrotic ILD). (**B**) Survival curves stratified by IPF diagnosis (IPF vs. non-IPF ILD). Statistical significance was assessed using the log-rank test. ***p < 0.001. IPF: idiopathic pulmonary fibrosis.
